# The Advent of CAR T-Cell Therapy for Lymphoproliferative Neoplasms: Integrating Research Into Clinical Practice

**DOI:** 10.3389/fimmu.2020.00888

**Published:** 2020-05-12

**Authors:** Marco Cerrano, Marco Ruella, Miguel-Angel Perales, Candida Vitale, Danilo Giuseppe Faraci, Luisa Giaccone, Marta Coscia, Molly Maloy, Miriam Sanchez-Escamilla, Hesham Elsabah, Afraa Fadul, Enrico Maffini, Gianfranco Pittari, Benedetto Bruno

**Affiliations:** ^1^Department of Oncology/Hematology, A.O.U. Città della Salute e della Scienza di Torino, Turin, Italy; ^2^Department of Molecular Biotechnology and Health Sciences, University of Torino, Turin, Italy; ^3^Department of Pathology and Laboratory Medicine, Abramson Cancer Center, University of Pennsylvania, Philadelphia, PA, United States; ^4^Adult Bone Marrow Transplantation Service, Department of Medicine, Memorial Sloan-Kettering Cancer Center and Weill Cornell Medical College, New York, NY, United States; ^5^Department of Hematological Malignancies and Stem Cell Transplantation, Research Institute of Marques de Valdecilla (IDIVAL), Santander, Spain; ^6^Department of Medical Oncology, Hematology/BMT Service, National Center for Cancer Care and Research, Hamad Medical Corporation, Doha, Qatar; ^7^Hematology and Stem Cell Transplant Unit, Romagna Transplant Network, Ravenna, Italy

**Keywords:** CAR T cells, adoptive immunotherapy, cellular therapy, lymphoma, leukemia

## Abstract

Research on CAR T cells has achieved enormous progress in recent years. After the impressive results obtained in relapsed and refractory B-cell acute lymphoblastic leukemia and aggressive B-cell lymphomas, two constructs, tisagenlecleucel and axicabtagene ciloleucel, were approved by FDA. The role of CAR T cells in the treatment of B-cell disorders, however, is rapidly evolving. Ongoing clinical trials aim at comparing CAR T cells with standard treatment options and at evaluating their efficacy earlier in the disease course. The use of CAR T cells is still limited by the risk of relevant toxicities, most commonly cytokine release syndrome and neurotoxicity, whose management has nonetheless significantly improved. Some patients do not respond or relapse after treatment, either because of poor CAR T-cell expansion, lack of anti-tumor effects or after the loss of the target antigen on tumor cells. Investigators are trying to overcome these hurdles in many ways: by testing constructs which target different and/or multiple antigens or by improving CAR T-cell structure with additional functions and synergistic molecules. Alternative cell sources including allogeneic products (*off-the-shelf* CAR T cells), NK cells, and T cells obtained from induced pluripotent stem cells are also considered. Several trials are exploring the curative potential of CAR T cells in other malignancies, and recent data on multiple myeloma and chronic lymphocytic leukemia are encouraging. Given the likely expansion of CAR T-cell indications and their wider availability over time, more and more highly specialized clinical centers, with dedicated clinical units, will be required. Overall, the costs of these cell therapies will also play a role in the sustainability of many health care systems. This review will focus on the major clinical trials of CAR T cells in B-cell malignancies, including those leading to the first FDA approvals, and on the new settings in which these constructs are being tested. Besides, the most promising approaches to improve CAR T-cell efficacy and early data on alternative cell sources will be reviewed. Finally, we will discuss the challenges and the opportunities that are emerging with the advent of CAR T cells into clinical routine.

## Introduction

Chimeric antigen receptors (CARs) are artificial proteins whose basic structure is composed of an antigen recognition ectodomain and an activation endodomain, linked by a spacer and a transmembrane domain (1). The ectodomain is a fusion protein encompassing single VH and VL regions of an immunoglobulin, joined by a linker peptide, i.e., a single-chain variable fragment (scFv), capable of non-MHC restricted surface antigen recognition (2–4). The endodomain is responsible for the intracellular signal transduction which follows T-cell activation and for the functional cytotoxic properties of the transduced cell, and its basic structure includes a CD3-derived component critical for transduction of activating signals triggered by a native T-cell receptor (TCR), predominantly a CD3ζ moiety containing immunoreceptor tyrosine-based activation motifs (ITAMs) (5).

CAR-based cellular immunotherapy was initially tested in patients with hematologic malignancies, and CD19 was selected as a preferential target antigen, based on its selective expression on B cells, therefore limiting *on-target off-tumor* side effects to B-cell aplasia, which may also protect against the risk of developing CAR-directed antibodies. Initial studies on autologous T cells engineered with anti-CD19 first-generation CARs demonstrated short effector persistence *in vivo*, despite multiple infusions (6). These suboptimal clinical outcomes were later circumvented by using autologous T cells engineered with upgraded CAR constructs, incorporating a co-stimulatory endodomain, typically CD28 or 4-1BB. These second-generation CAR constructs were intended to provide T cells with a supplementary activation signal upon ligand engagement, thus mimicking the physiologic second signal generated by CD28 independent binding to CD80/86 ligands following TCR antigen recognition. Alternative signaling from 4-1BB or CD28 in the context of second-generation CARs elicits non-overlapping pathways, resulting in T cells with remarkably distinct qualities. Specifically, CD28 generates a more rapid and intense co-stimulatory signal, compared to 4-1BB, in spite of a less durable cytotoxic activity and a shorter *in vivo* persistence of CAR T cells (7, 8).

Currently, two different second-generation anti-CD19 CAR T-cell products have been approved by US Food and Drug Administration (FDA) and by European Medicine Agency (EMA) for clinical use, but certainly further advancements are needed, in order to improve efficacy, broaden the spectrum of target diseases, and mitigate side effects. In addition, efforts are required to translate pre-clinical and early stage clinical research innovations into clinical practice.

## Major Clinical Studies Involving Anti-CD19 CAR T Cells

### Early Studies of CAR T Cells in Lymphoid Neoplasms

After the seminal studies of this unique form of adoptive T-cell therapy led by Eshhar and Goverman (9, 10), the breakthrough of CAR-based strategy emerged with the treatment of B-cell malignancies in the first decade of 2000s. Following the initial preclinical observations from Seattle Children's Hospital on the activity of first and second-generation constructs (11, 12), in 2010 Rosenberg and colleagues from National Cancer Institute (NCI) reported the first clinical response to an anti-CD19 CAR T-cell product in a patient with advanced follicular lymphoma (FL) (13). Shortly after, several early-phase studies confirmed the impressive anti-tumor effect of second-generation CAR T cells in heavily pretreated patients with B-cell malignancies, but also outlined the significant toxicities associated with this treatment, the most frequent being cytokine release syndrome (CRS) and neurotoxicity (NTX) (see below) (14–16).

The Memorial Sloan Kettering Cancer Center (MSKCC) group reported significant activity of their CD28 construct in B-cell acute lymphoblastic leukemia (B-ALL) in 5 R/R patients, all achieving a measurable residual disease (MRD) negative complete remission (CR) (17), although CRS was significant. Indeed, in keeping with observations in animal studies (12), T cells engineered with a CD19-specific second-generation CD28/CD3ζ dual-signaling CAR (CD19-28z) displayed superior *in vivo* persistence than first-generation ones, and resulted in favorable clinical responses in ALL and in patients with advanced B-cell Non-Hodgkin lymphomas (B-NHL) (18, 19). Another CD28 construct, KTE-C19 – now developed as axi-cel – designed at the NCI, was successfully employed in patients with refractory diffuse large B cell lymphoma (DLBCL) and indolent B-cell malignancies, showing a response in 12/15 cases, including 8 CR (18). Signs of CRS and/or NTX were observed in the majority of patients.

Similarly, T cells transduced with a anti CD19 CAR containing the 4-1BB and CD3ζ signaling domains (CD19-BBz) exhibited prolonged *in vivo* persistence and expansion, correlating with sustained clinical benefit in individuals with R/R B-ALL (16) and chronic lymphocytic leukemia (CLL) (14)^.^ Investigators of the University of Pennsylvania (UPenn), after showing the efficacy of their CD19-BBz construct CTL019 – now developed as tisa-cel – in 2 children with R/R B-ALL achieving CR (20), reported on a single-center phase I/IIa study on 30 R/R B-ALL patients. Morphologic CR was obtained in 90% of patients, and 6-month event-free survival (EFS) was 67%. All patients developed CRS, which was severe in 27% of the cases (16). In CLL, the same construct was tested in a single center pilot study including 14 heavily pretreated patients. Overall response rate (ORR) was 57%, and CR rate was 29%. Median duration of response was 40 months for CR patients, and CRS and NTX occurred in nine and five cases, respectively (14).

### Studies Leading to FDA-Approval of CAR T Cells

**Figure 1:**
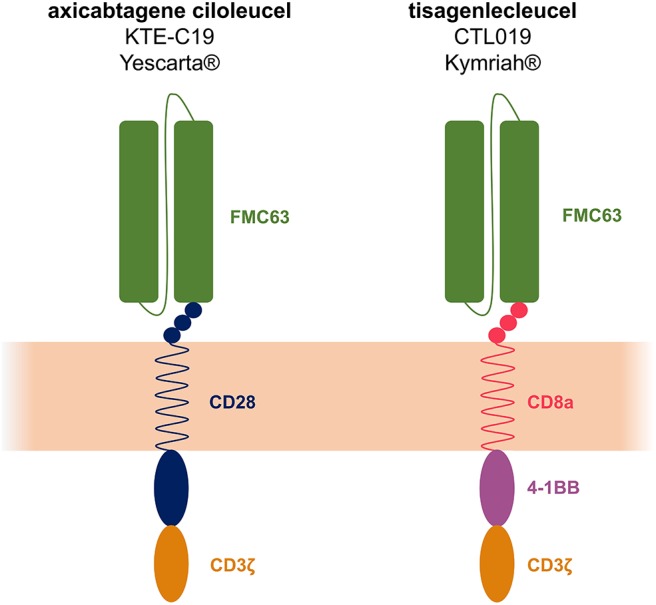
Axicabtagene ciloleucel (axi-cel or KTE-C19) and tisagenlecleucel (tisa-cel or CTL019) structure. The 2 constructs share identical recognition (FMC63-scFv) and signaling (CD3ζ) domains but the co-stimulatory domains differ: CD28 for axi-cel and 4-1BB for tisa-cel.

The FDA granted the autologous CAR T-cell therapy tisagenlecleucel (CTL019 or tisa-cel, KYMRIAH®, jointly developed by the UPenn and Novartis) breakthrough therapy designation for pediatric and young adult R/R B-ALL in July 2014. In August 2017 the FDA announced the approval of tisagenlecleucel for the treatment of R/R B-ALL for patients up to 25 years of age. An additional autologous anti-CD19 CAR T-cell therapy was FDA-approved in October 2017, i.e., axicabtagene ciloleucel (axi-cel, YESCARTA®, Kite Pharma Inc.) for adult patients with R/R DLBCL, primary mediastinal large B-cell lymphoma (PMBCL), high grade B-cell lymphoma and transformed FL. Soon after, tisagenlecleucel was also FDA-approved for adult with R/R DLBCL (of note, tisa-cel is not labeled for PMBCL). In 2018, the European Medicines Agency (EMA) approved the two constructs for similar indications as well. Tisa-cel and axi-cel CAR constructs share identical recognition (FMC63-scFv) and signaling (CD3ζ) domains but possess distinct co-stimulatory domains (i.e., 4-1BB and CD28 for tisa-cel and axi-cel, respectively, Figure 1).

**ELIANA (CCTL019-B2202)** was the first global CAR T-cell therapy registration trial that led to the FDA-approval of tisagenlecleucel for pediatric and young adults with R/R B-ALL (21). It was a multicenter single-arm phase II study enrolling a total of 92 patients. At enrollment, patients had received a median of three prior lines of therapy and 61% had failed a previous allogeneic hematopoietic stem cell transplantation (allo-HSCT). In the update analysis on 75 infused patients with a median follow-up of 13.1 months, best ORR was 81%, with 60% CR, all of whom MRD negative (<0.01% by flow cytometry). Tisa-cel was detected in blood up to 20 months after the infusion. Twelve months EFS and overall survival (OS) were 50 and 76%, respectively. Globally, 73% of the infused patients developed grade 3/4 adverse events (AEs) suspected to be related to tisagenlecleucel, and 47% of them were admitted to the intensive care unit (ICU) for CRS management. Neurological events were observed in 40% of the cases within 8 weeks after the infusion, 13% being of grade 3 but none of grade 4. Globally, 24% of patients were infused in the outpatient setting.

**ZUMA-1** was the first multicenter phase I/II clinical trial testing axicabtagene ciloleucel in aggressive B-NHL (22, 23), leading to the approval of the product. The phase II portion of the trial enrolled 119 patients, of whom 108 were infused and 101 evaluated for clinical response. Enrolled patients suffered from R/R DLBCL (76%), PMBCL (16%), or transformed FL (8%), and the majority of the cases had received at least three lines of chemotherapy before CAR T-cell infusion (69%); 26% had primary refractory disease. After lymphodepleting chemotherapy with low-dose cyclophosphamide and fludarabine, patients received CAR T cells at target dose of 2 × 10^6^ cells/kg. In this trial systemic bridging chemotherapy was not allowed after leukapheresis and the median time from leukapheresis to delivery of axi-cel to the treatment centers was 17 days. An updated analysis of the trial by Locke et al. showed a best ORR and CR rate of 83% and of 58% per investigators' assessment, respectively. Responses were durable, with 39% of patients maintaining the response at a median follow-up of 27.1 months, including 37% with persistent CR. Of note, CAR T-cell expansion after infusion was significantly associated with response. The median duration of progression-free survival (PFS) was 5.9 months, with PFS rates of 49% at 6 months and 41% at 15 months. Median OS was not reached, with an estimated 24-month OS rate of 50.5% and two responding patients underwent allo-HSCT. Globally, 95% of patients developed grade ≥3 AEs. A total of 93% of patients experienced CRS, 13% of grade 3/4, including a death related to hemophagocytic lymphohistiocytosis (HLH). NTX occurred in 64% of patients, 28% being grade ≥3 (22, 24, 25).

**JULIET (CCTL019-C2201)** was the multicenter single-arm pivotal phase II clinical trial that led to the FDA-approval of tisagenlecleucel for adult patients with R/R DLBCL (26). The trial enrolled 165 patients, of whom 111 were infused and 93 were evaluated for clinical response. Patients had received at least two prior lines of therapy and 54% had failed a prior autologous HSCT. The median time from enrollment to infusion was 54 days and the majority of patients (92%) received a bridging therapy to keep hematological disease under control. Patients received – after lymphodepleting chemotherapy in 93% of cases – a median CAR T-cell dose of 3.1 × 10^8^ (range 0.6–6.0). The median follow-up for data cutoff was 14 months. Best ORR was 52%, with 40% of CR and 12% of partial response (PR). Among patients with CR/PR as best response (*n* = 48), the relapse-free survival (RFS) was 65% at 12 months. At 12 months, OS was 40%. Grade 3/4 CRS rate was 22%, and globally 24% of the patients were admitted to the ICU for CRS management. NTX developed in 21% of patients, with 12% of the cases being of grade 3/4, without fatalities.

### Additional Studies on CD19-Targeting CARs in B-ALL and B-NHL

**ROCKET** was a multicenter single-arm phase II clinical trial testing the CD28 construct JCAR015, developed by Juno Therapeutics. The study enrolled 38 adult patients with R/R B-ALL and, after a median follow-up of 12.9 months, median EFS and OS were 4.4 and 8.1 months, respectively. CRS was observed in 27% of the cases and 29% of the patients experienced grade 3/4 NTX. Following several episodes of cerebral edema, some of which were fatal, the trial was halted by FDA. After a thorough analysis, investigators concluded that a surge in inflammatory cytokine levels from rapid, early T-cell proliferation may have provoked the blood–brain barrier (BBB) disruption seen in these patients, inducing fatal cerebral edema (27). However, the precise underlying cause remains unknown.

**TRANSCEND-001** was the first-in-human study testing the second-generation CD19-BBz product JCAR017 (lisocabtagene maraleucel, liso-cel), developed by Juno Therapeutics and characterized by a defined ratio of 1:1 CD8+ and CD4+ CAR T cells. This multicenter phase I clinical trial enrolled patients with R/R aggressive B-NHL, and the results of the DLBCL cohort (including also cases transformed from indolent lymphomas, high-grade B-cell lymphomas with MYC and BCL2 and/or BCL6 rearrangements, PMBCL and grade 3B FL) were presented at 2019 American Society of Hematology meeting. A total of 342 patients were leukapheresed and 268 ones received liso-cel, at 3 different dose levels. The dose of 100 × 10^6^ viable CAR T cells was chosen for dose confirmation and thus administered in the majority of patients (*n* = 176). Among the 256 heavily pretreated patients evaluable for efficacy, 34% of whom received a prior autologous-HSCT, ORR was 73%, with 53% of patients achieving a CR. After a median follow-up of 12 months, median duration of response (DOR) was not reached and PFS and OS were estimated at 6.8 and 21.1 months, respectively. In this large clinical trial, liso-cel demonstrated clinical activity across different subgroups, including patients with high-risk prognostic features. Regarding safety profile, CRS developed in 42% and NTX in 30% of patients, including 2% grade 3/4 CRS and 10% grade 3/4 NTX. Prolonged grade 3/4 cytopenias were reported in 37% of patients. Globally, seven deaths were recorded, 4 of which considered to be related to liso-cel (28).

**ZUMA-2** is a multicenter phase II trial which evaluated KTE-X19, a product which shares the same design of axi-cel but which is obtained in a different manufacturing process removing CD19-positive malignant cells, in patients with R/R mantle cell lymphoma (MCL). Seventy-four patients were enrolled, and the product was administered to 68 ones. ORR and CR rate were 93 and 67% in the primary efficacy analysis involving the first 60 infused patients, and 85 and 59% by *intention-to-treat*, respectively. After a median follow-up of 12.3 months, the estimated 12 months PFS and OS were 61 and 83% in the primary efficacy analysis populations. Cytopenias occurred in 94% of the patients and grade 3/4 CRS and NTX in 15 and 31% of the cases, respectively. Two grade 5 infectious adverse events were recorded (29).

**ZUMA-3** is a phase I/II trial evaluating KTE-X19 in adult patients with R/R B-ALL (NCT02614066) (30). Until now, only the phase I results have been reported. After a median follow-up of 16 months, 45 patients had received KTE-X19, 66% of which had received ≥3 prior lines of therapy. There were 2 reported KTE-X19–related grade 5 AEs (cerebral infarction and multiorgan failure), both in the context of CRS. CRS and NTX of grade ≥3 occurred in 29 and 38% of the patients, respectively. Of 41 patients with a follow-up ≥2 months, 68% achieved CR/CR with incomplete count recovery (CRi) and 73% reached undetectable MRD. With the limitation of small patient numbers and the presence of some confounding factors, prior blinatumumab treatment did not seem to jeopardize the manufacturing process or affect clinical results (31). The phase II of the trial is currently ongoing at the 1 × 10^6^ infusion dose and with revised adverse event management recommendations. Another phase I/II trial (ZUMA-4, NCT02625480) is evaluating axicabtagene ciloleucel in pediatric/adolescent patients with R/R B-ALL (32).

Mature data on an autologous CD19-28z CAR T-cell construct completely manufactured at **MSKCC** (NTC01044069) in relapsed B-ALL adult patients were reported by Park and colleagues (33). Fifty-three patients received CAR T cells and CR was obtained in 83% of the cases. After a median follow-up of 29 months, median EFS and OS were 6.1 and 12.9 months, respectively. Patients with <5% bone marrow blasts before treatment had an improved remission duration and survival, with median EFS and OS of 10.6 and 20.1 months, respectively. After infusion, severe CRS occurred in 14 patients and was fatal in one of them. Patients without morphological marrow remission before treatment, higher burden of disease or extra-medullary disease had a greater incidence of CRS, NTX, and shorter long-term survival.

The field of anti-CD19 CAR T-cell development in B-NHL and B-ALL is rapidly expanding, with several additional studies being recently reported (34–39), and more than 100 clinical trials currently registered worldwide. Comparing the results of these studies is becoming increasingly complex, given the differences in patient population, bridging therapy and toxicity grading system employed. Selected clinical trial results in B-ALL and B-NHL are summarized in Tables 1, 2.

**Table 1:** Selected clinical trials of CAR T cells in B-cell acute lymphoblastic leukemia.

**Setting**	**Target**	**Product**	**Costimul. domain**	**Generation**	**Vector**	**Population infused**	**Response**	**Durable remission rate**	**CRS (gr.3/4)**	**NTX (gr.3/4)**	**Institute/Company**	**Clinical trial**	**References**
B-ALL	CD19	Tisagenlecleucel (CTL019, Kymriah®)	4-1BB	Second (2nd)	Lentiviral	75, R/R pediatric/AYA B-ALL (up to 25 yo)	ORR 81% CR 60%	RFS 80% at 6 mo 59% at 12 mo	47%^¥^	13%^±^	UPenn/Novartis	ELIANA *NCT02435849* multicenter phase II	(21)
		KTE-X19	CD28	Second (2nd)	Retroviral	45, R/R adult B-ALL	ORR 82% CR 73%	Median DOR 12.9 mo	29%^§^	38%^±^	Kite, Gilead	ZUMA-3 *NCT02614066* multicenter phase I/II	(30)
						24, R/R pediatric/AYA B-ALL	CR + CRi rate 64–100% (related to CART dose)	ongoing remission 25–56% (related to CART dose)	22–75%^§^ (related to CART dose)	11–36%^±^ (related to CART dose)		ZUMA-4 *NCT02625480* Multicenter phase I/II	(32)
		19-28z-CAR	CD28	Second (2nd)	Retroviral	53, R/R adult B-ALL	CR 83%	Unknown	26%^¶^	42%^±^	MSKCC/Juno	*NCT01044069* Phase I	(33)
		CD19-28z-CAR	CD28	Second (2nd)	Retroviral	19, R/R pediatric/AYA B-ALL (up to 30 yo)	CR 67%	12-mo LFS 78.8%	28%^±^	5%^¬^	NCI	*NCT01593696* Phase I	(40)
		CD4^+^/CD8^+^ CD19-BBz-CAR	4-1BB	Second (2nd)	Lentiviral	45, R/R pediatric/AYA B-ALL	MRD^−^ CR 89%	Unknown	23%^±^	21%^±^	SCH	PLAT-02 *NCT02028455* Phase I/II	(41)
		CD19-BBz.EGFRt-CAR	4-1BB	Second (2nd)	Lentiviral	30, R/R adult B-ALL	CR 93% MRD neg 86%	Unknown	23%^§^	50%^±^	FHCRC	*NCT01865617* Phase I/II	(42)
		UCART19	4-1BB	Allogeneic CAR T cells, gene editing with TALEN	Lentiviral	7, R/R pediatric B-ALL	CR + CRi 88% MRD neg 86%	Ongoing remission 28%	15%^†^	0% ^†^	MDACC, UCL etc./Servier	PALL *NCT02808442* Phase I	(43)
						13, R/R Adult B-ALL		Ongoing remission 21%				CALM *NCT02746952* Phase I	
	CD22	CD22-BBz-CAR	4-1BB	Second (2nd)	Lentiviral	21, R/R pediatric/adult B-ALL	CR 73%	Median DOR 6 mo	0%^±^	0%^±^	NIH	*NCT02315612* Phase I	(44)

¥ , Penn/CHOP grading scale;

§ , NCI 2014 consensus grading scale modified by Lee DW et al. (45);

¶ , MSKCC criteria;

¬ , CTCAE v4.02;

± , CTCAE v4.03;

† *CTCAE v5.0*.

*ALL, acute lymphoblastic leukemia; R/R, relapse/refractory; AYA, adolescent and young adult; ORR, overall response rate; CR, complete remission; CRi, complete remission with incomplete hematologic recovery; MRD, minimal residual disease; mo, months; DOR, duration of response; EFS, event-free survival; RFS, relapse-free survival; LFS, leukemia-free survival; DFS, disease-free survival; NR, not reached; CRS, cytokine release syndrome; NTX, neurotoxicity; UPenn, University of Pennsylvania Hospital; SCH, Seattle Children's Hospital; FHCRC, Fred Hutchinson Cancer Research Center; MSKCC, Memorial Sloane Kettering Cancer Center; NCI, National Cancer Institute; MDACC, MD Anderson Cancer Center; UCL, University College of London*.

**Table 2:** Selected clinical trials of CAR T cells in B-cell non-Hodgkin lymphomas and chronic lymphocytic leukemia.

**Setting**	**Target**	**Product**	**Costimul. domain**	**Generation**	**Vector**	**Population infused**	**Response**	**Durable remission rate**	**CRS (gr.3/4)**	**NTX (gr.3/4)**	**Institute/Company**	**Clinical trial**	**Reference**
B-cell NHL	CD19	Axicabtagene ciloleucel (KTE-C19, Yescarta®)	CD28	Second (2nd)	Retroviral	108, R/R DLBCL, PMBCL, t-FL	ORR 83% CR 58%	Median DOR 11.1 mo	11%^§^	32%^±^	NCI/Kite, Gilead	ZUMA-1 *NCT02348216* multicenter phase I/II	(25)
		KTE-X19				68, R/R MCL	ORR 93% CR 67%	Unknown	15%^§^	31%^±^		ZUMA-2 *NCT02601313* multicenter phase II	(29)
		Tisagenlecleucel (CTL019, Kymriah®)	4-1BB	Second (2nd)	Lentiviral	111, R/R DLBCL, t- FL	ORR 52% CR 40%	12-mo RFS 65%	22%^¥^	12%^±^	UPenn/Novartis	JULIET *NCT02445248* Multicenter phase IIa	(26)
		Lisocabtagene maraleucel (JCAR017)	4-1BB	Second (2nd)	Lentiviral	269, R/R DLBCL, t-FL, PMBCL, FL3B, HGBCL	ORR 73% CR 53%	12-mo DOR 54.7%	2%^§^	10%^±^	FHCRC/Juno, Celgene	TRANSCEND-001 *NCT02631044* Multicenter phase I	(28)
		Lisocabtagene maraleucel (JCAR017) + Durvalumab	4-1BB	Second (2nd)	Lentiviral	11, R/R B-NHL	ORR 91% CR 64%	Unknown	Unknown	Unknown	Celgene/Juno	PLATFORM *NCT03310619* Phase I/II	(46)
		JCAR014 + Durvalumab	4-1BB	Second (2nd)	Lentiviral	15, R/R Aggressive B-NHL	ORR 50%	Ongoing remission 33%	7%^§^	0%^±^	FHCRC/Juno, MedImmune	*NCT02706405* Phase Ib	(47)
	CD20	CD20-CAR	None	First (1st)	Plasmidic	7, R/R Indolent NHL, MCL	ORR 43% CR 28%	Median DOR 5 mo	0%^°^	0%^°^	FHCRC	*NCT00012207* Phase I	(48)
		scFvFc.CD28-CD137z	CD28 4-1BB	Third (3rd)	Plasmidic	3, R/R indolent NHL, MCL	ORR 100% CR 67% PR 33%	Unknown	0%^°^	0%^°^	FHCRC	*NCT00621452* Phase I	(49)
CLL	CD19	Tisagenlecleucel (CTL019, Kymriah®)	4-1BB	Second (2nd)	Lentiviral	14, R/R CLL/SLL	ORR 57% CR 29%	Unknown	43%^¥^	7%^°^	UPenn/Novartis	*NCT01029366* Phase I	(14)
		Lisocabtagene maraleucel (JCAR017)	4-1BB	Second (2nd)	Lentiviral	23, R/R CLL/SLL	ORR 81% CR 45%	Unknown	9%^§^	22%^±^	Juno, Celgene	TRANSCEND-CLL-004 *NCT03331198* Phase I/II	(50)
		CTL119.BBz-CAR + Ibrutinib	4-1BB	Second (2nd)	Lentiviral	19, r/r cll	ORR 71% CR 43%	Ongoing remission 53% at 12 mo (MRD^−^ 37%)	16%^¥^	5%^±^	UPenn	*NCT02640209* Pilot trial	(51)
		CD19-BBz.EGFRt-CAR	4-1BB	Second (2nd)	Lentiviral	24, r/r cll Post-ibrutinib (5 richter, 25% post -venetoclax)	ORR 71% CR 21% MRD neg 58%	Unknown	25%^§^	25%^±^	FHCRC	*NCT01865617* Phase I/II	(52)

¥ , Penn/CHOP grading scale;

§ , NCI 2014 consensus grading scale modified by Lee DW et al. (45);

° , CTCAE v3.0;

± *, CTCAE v4.03*.

*NHL, non-Hodgkin lymphoma; DLBCL, diffuse large B-cell lymphoma; t-FL, transformed follicular lymphoma; FL3B, grade 3B follicular lymphoma; MCL, mantle cell lymphoma; PMBCL, primary mediastinal B-cell lymphoma; HGBCL, high-grade B-cell lymphoma; CLL/SLL, chronic lymphocytic leukemia/small lymphocytic lymphoma; R/R, relapse/refractory; ORR, overall response rate; CR, complete remission; PR, partial response; MRD, minimal residual disease; DOR, duration of response; OS, overall survival; PFS, progression-free survival; RFS, relapse-free survival; CRS, cytokine release syndrome; NTX, neurotoxicity; UPenn, University of Pennsylvania Hospital; FHCRC, Fred Hutchinson Cancer Research Center; NCI, National Cancer Institute*.

## CAR T-Cell Toxicity

### Cytokine Release Syndrome Diagnosis and Management

CRS is the most frequent AE associated with CAR T-cell therapy, being described in 50–90% of the patients in major clinical trials (21, 22, 24–26), and most commonly occurring within the first days after product infusion. CRS is a systemic inflammatory condition, which originates from direct activation and expansion of T cells after the interaction with target cells, leading to the production of cytokines such as TNF-alpha and INF-gamma. Besides, activated macrophages are the main responsible for IL-6 and IL-1 secretion, an essential event for CRS progression (53, 54). Furthermore, activated endothelial cells appear particularly important for the development of severe CRS.

CRS could range from a self-limiting flu-like syndrome to a life threatening multi-organ dysfunction, requiring immediate intervention and intensive life-supporting treatments. The CRS-associated capillary leak syndrome can lead to hypotension, reduced renal blood flow and pulmonary edema. Besides, it can be accompanied by clinical and/or laboratory evidence of macrophage activation, or even turn into a full-blown HLH (55), and specific criteria for the diagnosis of for CAR T-cell-related HLH/macrophage activation syndrome have been proposed (56). A reliable way to evaluate CRS is of utmost importance, in order to weight the incidence and the severity of CRS, but the comparison of different products and trials has been complicated by the lack of a uniform grading system (14, 45, 56). To overcome this issue, a consensus grading system for CRS and NTX associated with immune effector cell therapies has been recently proposed by the American Society for Transplantation and Cellular Therapy (ASTCT) (57).

Along with aggressive supportive measures usually in the contest of an ICU, severe CRS management requires the use of direct cytokine inhibition. The FDA-approved drug tocilizumab – an IL-6 receptor antagonist – is currently employed as first line treatment for CRS (usually ≥G2) and is also under investigation as a prophylactic strategy (58). For patients with an unsatisfactory response, second line relies on systemic corticosteroids. In this regard, recent data showed that early therapeutic intervention at the first signs of CRS – including the use of corticosteroids – can prevent serious complications without affecting clinical results, mitigating the fear that these drugs could hamper CAR T-cell efficacy (59, 60). Although in some centers corticosteroids are now commenced early, possibly at the same time as tocilizumab (61), the aforementioned results might not be valid for all CAR T-cell products, and the best dose and duration of corticosteroids treatment remains to be defined.

Additional cytokine antagonist agents (e.g., the anti-IL-6 siltuximab, the TNF inhibitor etanercept, and the anti-IL-1 receptor anakinra) (4, 56, 62) or ibrutinib, as suggested in a preclinical model (63), may represent further options in refractory cases. Recently, the tyrosine-kinase inhibitor dasatinib was shown to suppresses CAR T-cell cytotoxicity, cytokine secretion, and proliferation, thus suggesting a potential role of this drug in the treatment of CRS (64, 65).

A standardized CRS management strategy has been hard to define, due to different grading systems and CAR T-cell products tested in clinical trials, and proposing recommendations for the best approach in refractory cases is even harder, given the small number of patients who received each treatment and the incomplete understanding of the pathogenesis of CRS.

Finally, alternative strategies to optimize safety without compromising efficacy of CAR T-cell therapy, such as fractionated intrapatient dosing, are also being developed (66).

### Neurotoxicity Diagnosis and Management

NTX is the second most frequent serious AE, and it has been specifically defined with the term “CAR T-cell-related encephalopathy syndrome” (CRES) (56) or the broader “immune effector cell-associated neurotoxicity syndrome” (ICANS) (57). ICANS can occur in the contest of CRS, and severe NTX is more frequent in patients with severe CRS (67); however, the two events are not directly related, since neurologic symptoms often do not develop simultaneously, starting sometimes before CRS or even after CRS resolution (55).

Severe ICANS is characterized by endothelial activation and increased BBB permeability, leading to high concentration of inflammatory cytokines in the cerebrospinal fluid (CSF). Consequently, brain vascular pericyte stress and further secretion of endothelium-activating cytokines cause a further increase in BBB permeability, in a vicious cycle (67). Moreover, in a preclinical macaque model both CAR and non-CAR T cells could accumulate in the CSF and in the brain parenchyma, suggesting their direct role in NTX development (68).

ICANS usually manifests with impaired attention, language disturbance, confusion, and disorientation. Headache, tremors, agitation, hallucination, and aphasia can also occur. More rarely and in severe cases, seizures, motor weakness, increased intracranial pressure, and cerebral edema may be present. The more recent grading system of NTX employs a 10-point scoring system following the Immune Effector Cell-Associated Encephalopathy (ICE) assessment tool and it considers five main neurological domains (57).

Patients with severe ICANS should be managed aggressively and a multidisciplinary approach is often necessary, including neurologic consultation. Electroencephalogram, brain magnetic resonance, and CSF examination are important in the work-up of a suspected ICANS to rule out other causes of NTX (56, 67). Levetiracetam can be used as a prophylactic strategy to prevent seizures, beginning on the day of CAR T-cell infusion, or it can be started at the first sign of NXT. First line treatment for NTX usually consists of corticosteroids, mainly dexamethasone or high dose methylprednisolone. Tocilizumab is used when concomitant CRS occurs, but its role for NTX treatment is less clear, given its inability to cross BBB and its possible capability to increase IL-6 levels in CSF, reason why the anti-IL-6 drug siltuximab is emerging as an effective alternative (56, 69). Recently, granulocyte-macrophage colony-stimulating factor (GM-CSF) was shown to be an important player in the pathogenesis of NTX, and the humanized monoclonal antibody anti-GM-CSF lenzilumab was able to effectively prevent CD19-CAR-induced neuroinflammation and CRS in preclinical models (70). A clinical trial testing lenzilumab with axi-cel in R/R DLBCL will soon be open for enrollment (NCT04314843).

Albeit life-threatening in some instances, including a few fatal episodes (67), ICANS is completely reversible alike CRS in the vast majority of cases, and most patients have a self-limited course.

### Additional Side Effects

Infusion reactions are usually mild and tumor lysis syndrome (TLS) is uncommon but can occur in patients with high tumor burden: both should be managed according to standard guidelines (62). Prolonged B-cell aplasia is very common and it is a marker of CAR T-cell persistence (14). The resulting hypogammaglobulinemia can be corrected with intravenous immunoglobulin replacement therapy. Severe pancytopenia is extremely common and can last several weeks after CAR T-cell infusion, in up to 30% of the cases persisting beyond day 30. Late hematological toxicity has been associated with high grade CRS and a recent stem cell transplantation (71). Cytopenia can be managed with growth factors and antifungal prophylaxis can be considered for prolonged neutropenia. Especially for patients receiving fludarabine-containing lymphodepleting regimens, a prolonged prophylaxis for pneumocystis jirovecii pneumonia and herpes zoster reactivation is recommended (69).

### Clinical Risk Factors and Biomarkers for CAR T-Cell-Related Toxicity

CAR T-cell-induced CRS and NTX pathophysiology currently remains poorly understood and risk factors for toxicity development have been mostly defined in the context of human studies.

First, investigators tried to identify clinical factors promoting CAR T-cell expansion, since high CAR T-cell peak counts in patients with CD19-positive lymphoid malignancies have been consistently correlated with the development of severe CRS (16, 40, 72, 73) and NTX (22, 73). High pre-treatment tumor burden is a major driver of CAR T-cell *in vivo* propagation and B-ALL patients developing severe CRS had a significantly higher baseline bone marrow blast count compared to those who developed lower-grade or no CRS (16, 62, 74). Globally, trials including patients with ALL reported higher rate of severe CRS compared to NHL ones (21, 22, 26, 30, 33), and this may be due to higher tumor burden, different disease distribution and more proliferative nature of ALL compared to NHL (75). Similar to severe CRS, TLS, and severe neurotoxic effects secondary to CAR T-cell infusion have been found to prevalently occur in individuals with greater tumor burden (33, 41, 56, 67).

Besides, in two studies investigating the clinical effects of anti-CD19 CAR T-cell products with defined CD4:CD8 ratio in R/R B-ALL (42) and NHL (73), Turtle and coworkers observed superior CAR T-cell peak levels in individuals receiving higher (i.e., 2 ×10^6^ vs. 2 ×10^5^/kg) CAR T-cell dose, likely accounting for the correlation between numbers of infused CAR T-cell and severe toxicity (40, 41, 76). Notably, disease burden and CAR T-cell dose have a synergistic, positive effect on CAR T-cell expansion. Thus, adapting CAR T-cell dose to disease burden, rather than defining an individual fixed dose for all patients, might optimize efficacy and safety in each case.

The co-stimulatory domain also play an important role, since 28z CAR T cells show superior and more rapid expansion compared to BBz ones (55); besides, T cells expressing CARs with hinge and transmembrane domains from the CD8-alpha molecule release significantly lower levels of cytokines *in vitro* compared to those derived from CD28 (77).

Combined cyclophosphamide/fludarabine (Cy/Flu) conditioning also improves expansion and persistence of CAR T cells, as opposed to single-agent cyclophosphamide (42, 73), presumably due to its intensified depleting effect on recipient lymphocytes (78). Consistent with this hypothesis, Cy/Flu has been found to be an independent predictor of severe CRS and ICANS post-CAR T-cell infusion (67, 76, 78).

Several biomarkers have been correlated with CRS and/or NTX development, such as LDH, ferritin, CRP, inflammatory cytokines, GM-CSF, von Willebrand Factor, and angiopoietin 2 (reflecting endothelia activation) and lower platelet count, but a definitive predictive model is still lacking (22, 67, 76, 79–82).

## Improving CAR T-Cell Efficacy

### Mechanisms of Resistance to CAR T-Cell Therapy

Clinical experience with anti-CD19 CAR T cells has identified two main causes of resistance or disease relapse: (1) the presence or appearance of antigen-negative tumor cells, and (2) intrinsic characteristics of effector T cells which limit their function and efficacy (i.e., antigen-positive relapses) (83).

Approximately 30% of pediatric and young adult B-ALL patients treated with anti-CD19 CAR T cells relapse with a CD19-negative disease (21), and CD19 loss and downregulation has been observed in the NHL setting as well (84). Indeed, in ZUMA-1 trial 33% of the patients with available post-relapse samples showed loss of CD19 expression (85). It is still unclear whether these CD19-negative cells are present in the initial cancer and overgrow thanks to a selective advantage under the pressure exerted by T-cell therapy, or if they result from *de novo* mutations. The lack of CD19 surface expression may be due to mutations or alternative splicing events (86, 87), suggesting the possibility that in some patients the CD19 protein may be truncated, therefore lacking the epitope that is necessary to trigger recognition and killing of tumor cells by CAR T cells and CD19 detection by flow cytometry (88). Preclinical models demonstrated the impact of antigen-loss on the efficacy of CAR T cells also in the setting of solid tumors (89, 90). A recent communication by Ruella et al. highlighted a novel rare mechanism of resistance to CAR T-cell therapy, conferred by the accidental transduction of a single leukemic B cell with the anti-CD19 CAR gene during therapy manufacturing (91). The authors showed that the CAR, erroneously expressed on the surface of the neoplastic cell, directly bound CD19–co-expressed on the same cell–hiding it from recognition and conferring resistance to reprogrammed T cells. An “antidote” to deplete these resistant cells has been recently developed *in vitro* (92). A further mechanism of CD19-negative immune escape, particularly relevant in MLL-rearranged B-ALL, is lineage switch from lymphoid to myeloid leukemia, with consequent loss of expression of B-lymphoid lineage antigens (93, 94).

Alternatively, CAR T-cell treatment failure can be due to antigen-positive relapses, which often occur early after product infusion and which may be due several T-cell related mechanisms [reviewed in (95)]. First, poor expansion and early elimination of adoptively transferred CAR T cells have been almost invariably described in patients who are refractory to the treatment or who relapse in the first months after product infusion (16, 21, 22, 33). Also, reduced anti-tumor activity, anergy and early CAR T-cell exhaustion have been correlated to inferior response rates, especially in CLL (96). Finally, the presence of an immunosuppressive microenvironment can significantly impair the functionality of CAR T cells and represents a major obstacle, especially for their application in solid tumors (see below).

**Figure 2:**
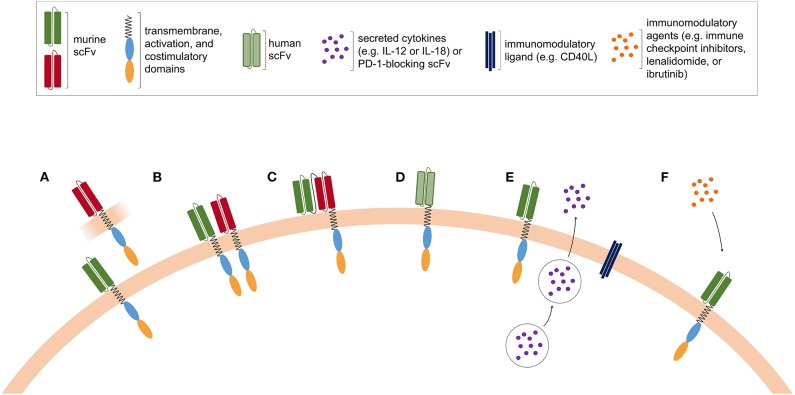
Possible strategies to improve CAR T-cell efficacy. **(A)** Pooled CAR T cells, consisting of two or more T-cell populations expressing CAR with distinct antigen specificities. **(B)** Dual CAR T cells are engineered to co-express CAR molecules with different antigen specificities. **(C)** Tandem CAR T cells express a bispecific CAR construct harboring two ligand-binding domains with different antigen specificities. **(D)** The murine-derived scFv is replaced in fully human CAR T cells, in order to limit the occurrence of immune rejection. **(E)** Armored CAR T cells are modified to secrete cytokines or to express immunomodulatory ligands together with the CAR. **(F)** CAR T cells can be co-administered with pharmacological agents with immunomodulatory properties.

Further improvement in lymphodepleting chemotherapy is currently in progress, with the aim of increasing the magnitude of CAR T-cell expansion and achieving more durable *in vivo* proliferation. In addition, the design of fully human CAR is under development to limit immune responses against murine-derived scFv and to prolong CAR T-cell *in vivo* persistence (97). Possible strategies to improve CAR T-cell efficacy are depicted in Figure 2.

### Targeting Alternative Antigens in Lymphoproliferative Diseases

A possible approach to overcome CD19 antigen loss is the targeting of alternative surface antigens, including CD20, CD22, immunoglobulin kappa (k) light chain, CD123, receptor tyrosine kinase-like orphan receptor (ROR1), and CD37, which are currently under evaluation for the treatment of B-cell malignancies (98).

After terrific results obtained with anti-CD20 monoclonal antibodies in B-cell lymphoproliferative diseases, anti-CD20 CAR T-cell therapy has been evaluated, showing enhanced persistence of transduced T cells – including both CD28 and 4-1BB co-stimulatory domains – and subsequent prolonged PFS in two of three treated patients with indolent B-cell lymphomas or MCL (48, 49).

Similarly to CD19, CD22 is also almost uniformly expressed on B cells, and it is currently under evaluation as a target in CD19-negative B-ALL relapses (99, 100). Data from a phase I trial including 21 children and adults with B-ALL infused with anti-CD22 CAR T cells showed a dose-dependent anti-leukemic activity. A total of 15 patients previously treated with anti-CD19 CAR T cells were included: CR was obtained in 73% of the cases, including CD19-negative ones, and median remission duration was 6 months (44). Interestingly, drug-induced CD22 up-regulation on leukemic cells could be a promising strategy to improve anti-CD22 CAR T-cell efficacy and remission durability (101).

Immunoglobulin k light chain antigen is another attractive target, although its expression is limited to a fraction of non-malignant B cells. On the other side, complete B-cell aplasia can be avoided, thus minimizing long-term humoral immunity impairment. Phase I clinical trials are evaluating efficacy and safety of anti-k light chain CAR T cells in B-NHL, CLL and multiple myeloma (MM) patients (102), with preliminary data showing no significant therapy-related toxicities.

ROR1 is a transmembrane glycoprotein crucial for cell proliferation and survival, constitutionally expressed on embryonal tissue and aberrantly on many adult malignant tissues, including B-cell malignancies (103–106). Humanized monoclonal antibodies, small molecule inhibitors, bispecific T-cell engagers (BiTE), and anti-ROR1 CAR T cells are under development (107–110).

Finally, preclinical studies showed that also anti-CD37 CAR T cells can be active against B-cell lymphomas (111, 112).

Despite some promising results, none of the antigens studied so far in B-cell malignancies appears to equal CD19, and the results of ongoing trials will probably clarify their role in future CAR T-cell development.

### Targeting Multiple Antigens in Lymphoproliferative Diseases

A reasonable strategy to successfully avoid antigen-negative relapses in hematologic tumors is to adoptively transfer CAR modified T cells targeting more than one tumor antigen. This goal may be achieved by administering to patients: (1) two mixed T-cell populations, each transfected with CARs showing different specificities (pooled CAR T cells, Figure 2A); (2) T cells that are engineered to co-express two CARs, each one competent to drive full T-cell activation (dual CAR T cell, Figure 2B); (3) T cells expressing one single CAR with two binding-domains in frame (tandem-CAR, Figure 2C) (113). Generally, tandem-CARs can induce activation of the CAR-expressing T cells by encountering either one of the two target antigens. Depending on the specific tandem-CAR construct, the binding of the CAR with the two antigens might have different effects on the downstream signaling and T-cell activation. Several pre-clinical studies (89, 90, 114, 115) have been published using bi- or tri-specific CARs, all showing that multiple-targeting is highly effective in preventing escape. Ruella et al. reported that targeting CD19 and CD123 on leukemic blasts represents an effective strategy for treating and preventing antigen-loss relapses occurring after CD19-directed therapies (116). Indeed, they devised a dual CAR-expressing construct that combined CD19- and CD123-mediated T-cell activation and demonstrated that it provides superior *in vivo* activity against B-ALL cells compared to single-expressing CARs or pooled CAR T cells. Other groups analyzed the efficacy of bispecific CAR T cells triggering robust cytotoxicity against target cells expressing either CD19 or CD20 (117, 118). Shah et al. recently reported data about a phase I first-in-human trial in R/R B-NHL exploring an anti-CD20/anti-CD19 bispecific CAR T-cell construct, with the aim of improving response rates and limiting CD19 negative relapse (118). Additional constructs co-targeting CD19 and CD22 are under development (119).

Globally, data on CAR T cells targeting more than one tumor antigen are still limited, but they are of extreme interest especially in settings in which CD19-negative relapses are more common or in which a specific target antigen is difficult to find (see also section Perspectives in Other Settings).

### Combination Therapies

Combining agents with different mechanisms of action may increase antitumor effect and reduce the risk of resistance (Figure 2F). As for normal T cells, CAR T-cell activity can be hampered by immune checkpoint proteins, such as programmed death ligand 1 (PD-L1), frequently expressed on tumor cells. Preclinical data have shown that combining CAR T cells with existing systemic checkpoint blockade antibodies potently enhances the eradication of established tumors (120–123). Thus, the combined use of immune checkpoint inhibitors with CAR T cells is currently being studied. The association of axi-cel plus atezolizumab is currently being tested in the ZUMA-6 trial (NCT02926833) with encouraging preliminary results (124) and two ongoing studies are evaluating JCAR014 (NCT02706405) (47) and liso-cel (NCT03310619) (46) in combination with another anti-PD-L1 antibody, durvalumab, in patients with relapsed/refractory B-NHL. Sequential approaches are being explored as well, and investigators at the UPenn recently started a phase I/II trial to evaluate the feasibility and efficacy of anti-PD-1 antibody pembrolizumab in patients failing to respond to (or relapsing after) tisa-cel therapy for B-NHL (NCT02650999) (125).

Besides, investigators are testing the co-administration of pharmacological agents with immunomodulatory properties, in order to revert immune dysfunctions that characterizes lymphoproliferative diseases, such as CLL and MM. In CLL, tumor cells contribute to generate several defects in innate and adaptive immune system function, which are predictive of a more aggressive disease (126–130). Long-term ibrutinib therapy exerts not only direct anti-tumor activity but also a valuable immunomodulatory effect on different immune cell compartments (131–134). Previous data had already shown that ibrutinib treatment enhances the generation of CAR T cells, and the co-administration of ibrutinib improved the engraftment and therapeutic efficacy of anti-CD19 CAR T cells in CLL and MCL mouse models (135, 136). The mechanisms underlying these ibrutinib-induced effects have not been completely clarified, and may be explained by *off-tumor* effects exerted on different immune compartments, particularly on T cells, but also by the tumor burden reduction which in turn may mitigate the immunosuppressive signals induced by the neoplastic clone. Based on these preclinical evidences, a prospective clinical trial combining humanized anti-CD19 CAR T cells with ibrutinib in CLL patients not achieving a CR after 6 months of single-agent ibrutinib treatment is currently ongoing at the UPenn (NCT02640209) (51). With similar intents, the administration of lenalidomide and other immunomodulatory drugs (IMIDs) in combination with CAR T cells is under evaluation in the setting of MM (137, 138). Preclinical data showed that lenalidomide combined with second-generation CAR T cells specific for the CS1 tumor antigen improves anti-myeloma properties and provided the basis to test this combination in the clinical setting (139). Newer agents are also being explored in order to improve expansion and persistence of CAR T cells, such as the 4-1BB agonist utomilumab, currently being tested in the ZUMA-11 trial with axi-cel (NCT03704298).

Combination therapies can also be used to target the tumor microenvironment, and anti-CD123 CAR T cells targeting both Hodgkin lymphoma cells and tumor-associated macrophages showed promising activity in preclinical models (140).

Oncolytic viruses (OV) have recently received considerable attention due to their potential ability to synergize with cancer immunotherapies. In different preclinical studies, the administration of armed-OV enhanced the immune functions of CAR T cells. OV expressing cytokines have been shown to enhance the survival of tumor-bearing mice by increasing CAR T-cell accumulation in the tumor and modulating its microenvironment (141, 142). Alternatively, the expression of a anti PD-L1 “minibody” by an OV could block the PD-1:PD-L1 interaction between CAR T cell and tumor cells, resulting in enhanced tumor control (143). Finally, release of BiTEs by an OV enhanced CAR T-cell homing and activation in the tumor, thus enhancing survival and improving efficacy (144, 145).

### *Armored* CAR T Cells

A possible strategy to increase CAR T-cell activity is to endow them with additional functions, aimed at improving tumor infiltration ability, effector functions, reduce immunosuppression, decrease tumor escape, or stimulate the native immune system. These novel engineered T cells are known as fourth-generation CAR T cells or armored CAR T cells (CAR T cells expressing an additional transgene for enhanced function, Figure 2E).

Commonly, fourth-generation CAR T cells are characterized by the secretion of specific cytokines, such as IL-12, IL-15, IL-18, and IL-21, and often referred as T cells redirected for universal cytokine-mediated killing or TRUCKS. IL-12 can improve T-cell cytolytic activity, mitigate T regulatory cell-mediated immunosuppression, and recruit and activate an innate immune cell response that can potentially avoid antigen-negative escape. As systemic administration of IL-12 was shown to cause severe side effects (146), constitutive or inducible expression of IL-12 has been integrated in CAR T cells (147–149). Expression of IL-15, either soluble or membrane-bound, improved survival and proliferation of CAR T cells without significant toxicity *in vitro* and in animal models (150–152). IL-18 is another cytokine included into TRUCKs and it was shown to improve CAR T-cell antitumor activity and increased NKG2D-positive NK cells tumor infiltration, while reducing the frequency of regulatory T cells and suppressive macrophages in the tumor micro-environment (153–155). Lastly, in a preclinical lymphoma model, IL-21 was shown to foster the generation of anti-tumor T cells with enhanced Wnt/β-catenin pathway and stem-like properties, thus potentially leading to long-lived memory CAR T cells (156). Alternatively, some of the effects induced by cytokines can be obtained by including a cytokine signaling domain for IL-2Rβ in the second-generation CAR construct (157).

Moreover, T cells can be redirected with additional domains to convert immunosuppressive signals into activating ones. For instance, dominant negative (DN) mutations of the TGF-β receptor in Epstein–Barr virus-specific T cells make them resistant to tumor-derived TGF-β in a lymphoma model (158). Another strategy to overcome the immune suppressive signals from tumor microenvironment is the inclusion of the so-called “switch receptors.” For example, the fusion of the extracellular domain of the IL-4 receptor with the intracellular one of the IL-7 receptor results in the activation and proliferation of T cells in the presence of the normally inhibitory cytokine IL-4 (159, 160). Similarly, a PD-1/CD28 chimeric switch receptor can convert the PD-L1 immunosuppressive stimulus into a costimulatory signal (161). Armored CAR T cells can also be modified to express ligands for costimulatory molecules. For instance, the expression of CD40L on CAR T cells leads to the activation of endogenous antitumor immune response (162) while the expression of 4-1BB-L increases the persistence of CD28-costimulated CART19 in B-ALL preclinical models (163). A clinical trial exploring armored CAR T cells in NHL and CLL is currently undergoing at MSKCC (NCT03085173) and preliminary results are promising (164). Additional effector functions can be obtained by the inclusion of chemokine receptors and ligands in armored CAR T cells. For example, IL-7 and CCL19 expression in CAR T cells was shown to improve their survival and activity in a solid tumor mouse model (165). Anti-CD30 CAR T cells expressing the chemokine receptor CCR4 showed better lymphoma infiltration and overall anti-tumor activity in preclinical models of Hodgkin lymphoma (166). Furthermore, anti-PD-L1 antibodies can be released by CAR T-cell within the tumor microenvironment, improving T-cell infiltration and functionality (167). An alternative and even more innovative approach has devised the generation of armored CAR T cells locally secreting PD-1-blocking scFv (168), thus avoiding potential toxicities associated with systemic checkpoint inhibition.

Furthermore, armored CAR T cells can secrete bispecific T-cell engagers or BiTEs. This technology allows targeting multiple tumor antigens together with the recruitment of tumor-infiltrating T cells. T cells that secrete BiTEs targeting tumor antigens such as EPHA2 (169), CD123 (170), and CD19 (171) are being developed.

### Modulating CAR T-Cell Regulation

The field of engineering T cells, including gene editing and suicide genes strategies (172), is rapidly evolving and it will likely allow to increase the potency and safety of CAR T cells. T-cell exhaustion is an important factor limiting antiviral and antitumor responses in the setting of chronic antigen exposure (173, 174), and it also contribute to reduce CAR T-cell efficacy. The structure and the regulation of CAR expression on the surface of engineered T cells play a central role in predisposing CAR T cells to chronic activation and exhaustion (7). In particular, antigen-independent signaling has been found to drive early exhaustion of CAR T cells and to limit their antitumor efficacy *in vivo* (7, 175), and it can be influenced by the type and the position of the co-stimulatory domain, the spacer length, and/or the promoter or the vector used to express the CAR. Therefore, in order to prevent or delay CAR T-cell dysfunction, several efforts are being made to optimize the design and the expression of CAR constructs. Eyquem et al. reported on an innovative approach targeting a CAR coding sequence to the TCR locus and placing it under the control of endogenous regulatory elements (176). This method reduces tonic signaling, averts accelerated T-cell differentiation and exhaustion, and increases the therapeutic potency of engineered T cells. More recently, Viaud et al. demonstrated in a syngeneic lymphoma murine model that their “switchable” CAR T-cell platform, which can incorporate “rest” phases through cyclical dosing of the switch, was able to induce a robust central memory population and enhance CAR T-cell expansion and efficacy (177). These findings can have several clinical implications, since the engineering of more functional T cells might reduce the T-cell doses employed, therefore eliciting milder toxicities (172).

## Emerging CAR T-Cell Applications

### Chronic Lymphocytic Leukemia

In recent years, CLL treatment paradigm has been revolutionized by B-cell receptor and BCL2 inhibitors (178). Nevertheless, relapses occur, especially in high-risk setting, such as patients with unfavorable genetic markers like *TP53* mutations. CAR T-cell treatment could represent a viable option in case of treatment failure, or might directly compete with targeted therapies–especially in patients with older age and unfavorable disease features, avoiding a long-term drug administration which can lead to toxicities (179), lack of compliance and ultimately cost problems (180).

Albeit CLL was one of the first diseases in which CAR T cells were used (15), experience is more limited compared to B-ALL or DLBCL. Nevertheless, safety and efficacy data are encouraging, especially in high risk patients (180).

The two FDA-approved anti-CD19 CAR T-cell constructs and JCAR017 are under investigation for R/R CLL (14, 50, 181). Turtle and colleagues reported on 24 CLL patients, the vast majority (92%) of whom had failed ibrutinib, treated with CD19-BBz CAR T cells after receiving lymphodepleting chemotherapy. Four weeks after infusion, CR+PR rate was 71%. Twenty patients (83%) developed CRS and eight developed NTX, with a fatal outcome in one patient. Fifty-eight percent of the patients who had deep sequencing of bone marrow samples after therapy reached MRD negativity. These data demonstrated that CAR T cells can be effective in CLL patients who failed targeted therapies, but longer follow-up is needed in order to evaluate the durability of such responses (52).

Thus far, remission rates obtained with CAR T cells in CLL are lower compared to B-ALL and DLBCL (14, 52, 96). Besides, responses appear to be weaker in the lymphnodes than in the bone marrow. Immune dysregulation typical of CLL may partly explain the lower efficacy of CAR T cells in these patients. Indeed, an impaired immune system (i.e., CD8^pos^ T cells with low proliferative and cytotoxic capacities, and/or less expansion of “naïve” CD4^pos^ T cells) can lead to decreasing CAR T-cell activation after transduction (182, 183). These intrinsic characteristics of CLL milieu are usually present at the time of diagnosis but are also favored by previous lines of treatment, particularly by fludarabine. These data could support the development of allogeneic CAR T cells from healthy donors, in whom the activity and cytotoxicity of T cells are not modified by the tumor clone (see section Alternative Sources) or the association with immunomodulatory agents, as discussed *supra*. Recent results of a pilot study evaluating CD19 CAR T cells concomitantly to ibrutinib in CLL (*n* = 19) showed a good tolerability, with robust CAR T-cell expansion and decreased CRS severity as compared to CLL patients treated with CAR T cells without ibrutinib, and similar response rates and long-term outcomes (184).

Alternative antigenic targets other than CD19 are under investigation in CLL, such as clonal light chain (kappa or lambda), CD23, the receptor of the invariant fragment of IgM (FcγR), and ROR1 (102, 107, 185, 186). Selected clinical trial results in CLL are summarized in Table 2.

### Multiple Myeloma

Recently, clinical and basic research on CAR T-cell treatment for MM has started to yield encouraging results and, among the MM-specific targets current under investigation, B-cell maturation antigen (BCMA) seems to be the most promising one (187). Indeed, two phase I clinical trials, conducted, respectively, at the NIH (NCT02215967) (188) and UPenn (NCT02546167) (189), showed remarkable results in preliminary reports.

Data about a third phase I multicenter trial (NCT02658929) exploring the second-generation CAR bb2121, composed of anti-BCMA scFv and a 4-1BB costimulatory domain, were recently published (190). The first 33 highly pretreated patients who received a bb2121 infusion showed an ORR of 85%, with a CR rate of 45%. A dose-dependent effect on the frequency and duration of response was observed: VGPR or better were observed only with doses ≥150 ×10^6^ CAR T cells. Median follow-up was 11.3 months and median duration of response was 10.9 months. CAR T-cell expansion was associated with response, and CAR T cells persisted up to 1 year after the infusion. Median PFS was 11.8 months, comparing favorably with other salvage therapies for a similar population (191, 192). CRS and NTX were reported in 76 and 42% of the patients, respectively, and were mostly mild. Phase II and III multicenter studies evaluating the efficacy and safety of bb2121 in subjects with R/R MM are currently ongoing in the US and in Europe (NCT03361748 and NCT03601078).

Results of another phase I trial in R/R MM patients employing LCAR-B38M, a bispecific CAR T-cell product that binds BCMA at two separate antigenic epitopes, were recently reported (193). After lymphodepletion based on cyclophosphamide, LCAR-B38M CAR T cells were administered in three separate infusions. At data cutoff, 57 patients received the product. CRS occurred in 90% of patients, 7% being grade ≥ 3 and one patient reported severe NTX. The ORR was 88%, including 68% of CR and MRD negativity was achieved by 63% of the patients. After a median follow-up of 8 months, median PFS was 15 months.

Although associated with high ORR, the main problem of anti-BCMA CAR T cells is the relatively short durability of responses, possibly due to the loss or down regulation of BCMA expression on MM cells and CAR T-cell limited persistence or functional exhaustion (194).

In addition to BCMA, alternative target antigens expressed on MM cells surface and compound CART cells expressing two (or more) different CARs are under investigation. Yan et al. explored the activity and safety of a combination of humanized anti-CD19 and murine anti-BCMA CAR T cells in patients with R/R MM (195). Twenty-one patients were infused and, after a median follow-up of almost 6 months, 95% of patients had an objective response, including nine stringent CR. The most common adverse event was CRS (90% of the patients), including 14% grade 3/4 cases. Ongoing studies are investigating several other potential targets on MM plasma cell surface (196), including CD44v6 (197), Lewis Y (198), NKG2D ligands (199), CD229 (200), and integrin β7 (201). Selected clinical trial results in MM are summarized in Table 3.

**Table 3:** Selected clinical trials of CAR T cells in multiple myeloma.

**Setting**	**Target**	**Product**	**Costimul. domain**	**Generation**	**Vector**	**Population infused**	**Response**	**Durable remission rate**	**CRS (gr.3/4)**	**NTX (gr.3/4)**	**Institute/Company**	**Clinical trial**	**References**
MM	BCMA	BCMA.CAR	CD28	Second (2nd)	Retroviral	16, R/R MM	ORR 81% ≥VGPR 63%	Unknown	38%^§^	6%^¬^	NIH	*NCT02215967* Phase I	(188)
		CART-BCMA	4-1BB	Second (2^nd^)	Lentiviral	25, R/R MM	ORR 48%	Median DOR ≈ 4 mo	32%^¥^	12%^*^	UPenn/Novartis	*NCT02546167* Single-center, phase I	(189)
		bb2121	4-1BB	Second (2nd)	Lentiviral	33, R/R MM	ORR 85% CR 45%	Median DOR 10.9 mo	6%^§^	3%^±^	NIH/Bluebird Bio, Celgene	*NCT02658929* Multicenter phase I	(190)
		LCAR-B38M	None	First (1st)	Lentiviral	57, R/R MM	ORR 88% CR 68%	Median DOR 14 mo	7%^§^	2%^±^	Nanjing Legend Biotech	LEGEND-2 *NCT03090659* Multicenter phase I/II	(193)
	k-Ig LC	κ. CAR	CD28	Second (2nd)	Retroviral	7, R/R MM	≥PR 57%	Unknown	0%^*^	0%^*^	BCM	CHARKALL *NCT00881920* Phase I	(102)
	NKG2DL	CM-CS1 T	CD3ζ plus DAP10	Second (2nd)	Retroviral	5, R/R MM	–	–	–	–	DFCI/Celyad	*NCT02203825* *in vitro* Phase I	(199)

¥ , Penn/CHOP grading scale;

§ , NCI 2014 consensus grading scale modified by Lee DW et al. (45);

* , CTCAE v4.0;

¬ , CTCAE v4.02;

± *, CTCAE v4.03*.

*MM, multiple myeloma; R/R., relapse/refractory; ORR, overall response rate; CR, complete remission; PR, partial response; VGPR, very good partial response; SD, stable disease; wk, weeks; EFS, event-free survival; DOR, duration of response; PFS, progression-free survival; CRS, cytokine release syndrome; NTX, neurotoxicity; UPenn, University of Pennsylvania Hospital; DFCI, Dana-Faber Cancer Institute; NIH, National Institutes of Health; BCM, Baylor College of Medicine*.

### Perspectives in Other Settings

In R/R Hodgkin lymphoma, CD30-directed CAR T-cell therapy showed a manageable safety profile and significant activity in phase I clinical trials, employing either CD30-BBz (202) or CD30-28z CAR T-cell products (203), especially after lymphodepleting chemotherapy was introduced (204). Nevertheless, the number of patients treated so far is small. Furthermore, CD30-directed CAR T cells are being evaluated in CD30-positive anaplastic large cell lymphoma, with encouraging results (202, 203).

In T-cell neoplasms, the development of CAR T cells been limited by the difficulty of targeting malignant cells without killing the very effector cells (fratricide) (205). Thus, several efforts have been made to find tumor-specific antigens (e.g., CD1a), which would prevent prolonged T-cell aplasia as well (206–208). Besides, fratricide killing could be avoided by knocking-out the target gene on effector cells using gene editing approaches (e.g., TALEN or CRISPR/Cas9), with some preclinical experience on CD7-knockout anti-CD7 CAR T cell already published (206, 209). Finally, CAR NK and allogeneic CAR T cells are being tested, with the advantage of avoiding a potential contamination of the product with malignant T cells and, for NK, also fratricidal killing (206) (see section Alternative Sources).

The development of CAR T cells for advanced solid tumors and relapsed/refractory AML, still associated with unfavorable prognosis despite recent therapeutic developments (210), has been limited by the absence of a suitable tumor-specific antigen (211, 212) and severe and toxicities due to on-target off-tumor effects occurred in some trials (213–217). Thus, investigators are trying to find more suitable targets (218, 219) and exploring strategies to promptly induce CAR T-cell exhaustion only when needed (220), such as mRNA electroporation (221, 222) and inducible suicide genes (223). CARs co-targeting two or three antigens are also being developed, aiming at preventing possible off-tumor side effects but also at avoiding antigen escape risk (115, 224–229). In solid malignancies, tumor microenvironment represents an unique obstacle which can significantly hamper CAR T-cell efficacy (230). Thus, innovative strategies such as CAR T-cell local delivery (231–234) and PD-1/PD-L1 axis block are being explored (120, 121, 235, 236).

A detailed description of the limited clinical results available so far has been recently reviewed elsewhere (212, 237).

## Alternative Sources

### Allogeneic CAR T Cells

Because of manufacturing issues, especially in heavily pre-treated patients, CAR T-cell production sometimes fails or requires too long; thus, universal allogeneic anti-CD19 CAR T-cell products obtained from healthy donors (*off-the-shelf* products) could represent a readily available solution. However, allogeneic T cells have very high alloreactive potential, because of TCR natural reaction against non-autologous tissues. Recently, thanks to gene editing technology, researchers succeeded in preventing the expression of endogenous TCR knocking down the TRAC gene (i.e., the gene codifying α chain of TCR), in order to minimize graft-versus-host disease (GVHD) risk in non-HLA matched recipients. As a matter of fact, TRAC loci can be disrupted using electrotransfer of mRNA codifying various nucleases (238–240), such as zinc finger nucleases (ZFN) (241), transcription activator-like effector nucleases (TALEN), megaTAL nucleases (242–244), and CRISPR/Cas9 systems (245).

UCART19 (a CD19-BBz product) is modified to lack both CD52 expression and the endogenous TRAC locus, and to include a RQR8 marker-suicide gene as “safety switch.” In this way, these allogeneic CAR T cells become resistant to anti-CD52 monoclonal antibody alemtuzumab, used for lymphodepletion along with cyclophosphamide and fludarabine to increase CAR T-cell persistence, and precautionary targeted elimination through anti-CD20 monoclonal antibody rituximab becomes possible. A phase I pediatric trial (PALL, NCT02808442) for high-risk R/R CD19-positive B-ALL and a phase I dose-escalation adult trial (CALM, NCT02746952) for patients with R/R B-ALL are underway. Preliminary data were presented at 2018 American Society of Hematology meeting (43). A total of 20 patients received at least one UCART19 infusion, 13 in CALM and 7 in PALL trial. After UCART19 infusion, 88% of evaluable patients (14/16) achieved CR or CRi, and 86% (12/14) of them reached MRD negativity. Globally, 11 patients underwent allo-HSCT. Preliminary data suggested that anti-leukemic activity was linked to CAR T-cell expansion. Severe CRS was reported in 15% of the patients and no severe NTX occurred. Grade 1 acute GVHD was reported in two patients.

In MM, allogeneic anti-BCMA CAR T cells induced sustained antitumor responses in mouse models and, importantly, maintained their phenotype and potency after scale-up manufacturing, standing promisingly for clinical evaluation (246).

Despite the reduced GVHD risk of TCR-negative *off-the-shelf* CAR T cells, these cells are exposed to killing by the patient's own mismatched T cells, leading to rejection and subsequently short-lasting response. Therefore, several attempts are under investigation to protect allogeneic CAR T cells from rejection, such as the use of ZFN gene-editing technology to eliminate HLA molecule expression from CAR T cells (247). Moreover, investigators are also trying to prevent NK activation, a possible cause of CAR T-cell rejection due to “missing self” recognition, enforcing expression of non-classical HLA molecules (i.e., HLA-E and HLA-G) (247–250).

An alternative approach – borrowed from regenerative medicine – is the generation of tumor-targeting T cells from induced pluripotent stem cells (iPSC), allowing to exploit the unlimited proliferative capabilities of iPSC together with the CAR-directed antigen specificity. Themeli et al. generated CD19-CAR-expressing T cell-derived iPSC with an effector memory phenotype, which were effective in killing CD19-positive lymphoma cells (251). Among the products currently under development for clinical application, FT819, an off-the-shelf iPSC product, is engineered to express a CD19-CAR together with the antibody-engaging CD16 Fc receptor (allowing dual-targeting), and to eliminate the TCR surface expression. FT819 was evaluated in the pre-clinical setting, resulting effective in targeting tumor cells both *in vitro* and *in vivo* in a mouse model of ALL (252). However, before moving to human studies, the risk of transferring undifferentiated iPSC with tumorigenic potential and the issue of host rejection need to be carefully addressed.

### CAR NK Cells

Another potential source of CAR carrier for *off-the-shelf* products is represented by NK cells. NK cells, which constitute approximately 10% of circulating lymphocytes, are efficient immune effector cells with a recognized role in the control of neoplastic proliferation (253). The interest toward these cells in this setting depends on their specific characteristics. In addition to the CAR-specific target recognition, transduced NK cells retain the ability to recognize neoplastic cells through their innate receptors, thus reducing the risk of tumor escape (254). Since NK cells do not require HLA matching for target recognition, allogeneic NK cells do not cause GVHD. Also, the limited life-span of NK cells reduces the risk of long term side effects.

Allogeneic NK cells can be derived from donor peripheral blood, bone marrow, or even from cord blood. Indeed, cord blood represents an optimal source, since NK cells can be readily available and can usually be collected in clinically relevant doses for adoptive immunotherapy (255). NK cells engineered to express a CD19-directed CAR, to ectopically produce IL-15 as a support for survival and proliferation, and to express a suicide gene (i.e., inducible caspase-9) showed killing ability *in vitro* and *in vivo* in a mouse model (256). The preliminary results of a trial exploring the safety and efficacy of CD19-28z-2A-iCasp9-IL15 transduced cord blood NK cells in patients with relapsed or refractory CD19-positive B-lymphoid malignancies (NCT03056339) have been recently published by Rezvani group. After lymphodepleting chemotherapy, 11 patients received a single infusion of CAR-NK. Eight of the 11 treated patients rapidly responded (within 30 days), 7 of whom achieving CR and no major side effect was recorded, including CRS, NTX, or GVHD (257).

Cell lines represent an alternative NK cell source, and among them NK-92 is the most widely used. NK-92 was established from a patient with NK-cell lymphoma, and is characterized by an activated NK-cell phenotype and a strong cytotoxic activity which, however, may not be sufficient to kill clonal cells of lymphoid origin (258, 259). CD19-CAR construct can be effectively expressed on NK-92 cells, conferring cytolytic capability toward previously resistant CD19-positive neoplastic cells *in vitro* and in mouse models (260–262). However, before *in vivo* application, cells need to be irradiated to avoid tumor engraftment, possibly negatively impacting on their efficiency.

A phase I/II trial evaluating PCAR-119 (NK-92 cell line engineered to express a CD19-TCRzeta-CD28-4-1BB CAR) in patients with CD19-positive lymphoproliferative diseases is currently open in China (NCT02892695), but results are not available yet. The result of the first-in-man clinical trial of CAR NK-92 cells have been reported in the setting of AML, where three patients were treated with a CD33 CAR NK-92, demonstrating the feasibility of the procedure (263).

## Integration of CAR T Cells Into Clinical Routine

### FACT Standards for Immune Effector Cells

Since 1966, the Foundation for the Accreditation of Cellular Therapy (FACT) has promoted quality practice in HSCT. More recently, FACT has broadened its objectives to also include standards in the nascent field of cellular therapies. In this context, FACT recently published the first edition of the Standards for Immune Effector Cells (IEC). These Standards apply to immune effector cells used to modulate an immune response for therapeutic intent, such as dendritic cells, natural killer cells, T, and B cells. This includes, but is not limited to, CAR T-cell therapy and vaccines (264).

Requirements for programs that administer immune effector cells in a center that is not already FACT-accredited are fully contained in the FACT IEC Standards. When IEC are administered in a FACT-JACIE accredited stem cell transplantation unit, the program must fully comply with the new 7th edition of FACT-JACIE Hematopoietic Cell Therapy Standards (265).

### Treating Site Preparation and Logistics

Each institution that initiates a CAR T-cell therapy program will face both clinical and administrative challenges before offering this new therapy to patients. This process involves complex logistics that cover the collection of cells at the apheresis center, shipping them to the manufacturer for production, coordinating receipt of the product, and defining an ideal workflow for CAR T-cell administration and patient management (266–269).

Hospitals need to develop a CAR T-cell consultation service to facilitate patient selection and treatment. Ideally, this service should consist of a multidisciplinary team that includes physicians, nurses, and social workers with expertise in cellular therapies, and an effective administrative infrastructure that ensures the execution of the workflow. There should be open communication between the center's and manufacturing site's staffs regarding any questions related to the timing of delivery and product quality. This multidisciplinary team, including physicians with expertise in CAR T cells and different medical specialists, should have regular meetings to review each patient's treatment course, and to develop institutional guidelines that include algorithms for the management of expected adverse events, such as CRS and NTX, and recommendations for the clinical staff of different departments (e.g., pharmacy, emergency department, neurology and intensive care unit). This is in accordance with Risk Evaluation and Mitigation Strategy (REMS) programs to reduce the potential risks of CAR T-cell agents, which require authorized centers to comply with specific guidelines, including the training of providers who prescribe, dispense, and administer CAR T cells. REMS programs also mandate patients and caregivers' education about toxicities, including a REMS wallet card given to each patient which reports the key symptoms possibly related to CAR T-cell therapy and the contacts of the referent physicians (270).

In many centers in the U.S., transplant programs have assumed this responsibility as they have clinical expertise in the diseases currently being treated with CAR T cells, and they are also well-versed in the logistics and regulatory aspects of delivering cellular therapies. However, some centers have developed other models, including self-standing immune effector cells services, or decided to deliver CAR T cells in disease-specific services.

Finally, community oncologists play a critical role, recognizing potentially eligible patients and following them after CAR T-cell treatment has been administered. Thus, to ensure optimal long-term outcomes for patients, it becomes essential to establish a prolonged communication between the reference center and the local oncologist, who must be familiar with common toxicities and their monitoring and management (271).

### CIBMTR Guidelines for Cellular Therapies

Data registries of HSCT recipients across Europe and United States have been essential to evaluate long-term follow-up results in this field. During the summer of 2016, the United States based Center for International Blood and Marrow Transplant Research (CIBMTR) launched the new Cellular Therapy Registry forms. These forms, based on the design of the transplant forms, aim to collect information on all cellular therapies including CAR T cells. The goal of the CIBMTR Cellular Immunotherapy Data Resource (CIDR) is to centralize cellular therapy data collection and reporting requirements such as the United States FDA follow-up obligation for genetically modified cells.

Additionally, in February 2018, the European Medicines Agency (EMA) workshop, including members from the European Society for Blood and Marrow Transplant (EBMT) and from the CIBMTR, reported their immediate priorities on patient data registries on CAR T-cell therapy (272). Specifically, they aimed to harmonize data element definitions across registries, to establish measures that ensure data are collected systematically with appropriate verification and quality assurances, to ensure arrangements are in place to permit data sharing, and to improve communications between registry holders, regulators and marketing authorization holders and applicants. The report also included recommendations to facilitate and improve registry data use including the systematic collection of a set of core commonly-defined data elements (273).

## Discussion

The advent of CAR T-cell treatment is revolutionizing established oncology paradigms, especially – to date – in B-cell malignancies. CAR T cells have already entered the treatment armamentarium for B-ALL and aggressive B-NHL, and the first presented real world data are reassuring and globally confirmed results of clinical trials (274–276), showing efficacy even beyond current indications (37, 277). The preclinical and clinical research field is extremely active, and we can anticipate an increase in treatment indications and available products in the near future. Indeed, the exciting results of ZUMA-2 trial in R/R MCL (29) open new possibilities in this difficult lymphoma subtype, and tisa-cel (ELARA trial, NCT03568461) and axi-cel (ZUMA-5 trial, NCT03105336) are being evaluated in R/R indolent lymphomas as well. Furthermore, the promising data on CAR NK and allergenic CAR T cells are opening the road to *off-the-shelf* products (43, 257).

CAR T-cell results are still hard to compare to those obtained with conventional treatment options, especially for the oldest trials lacking of data by *intention-to-treat* and considering the heterogeneity of treated patients [e.g., B-ALL patients with MRD positivity and in full blown relapse (33) included in the same trials]. Long-term outcomes are of extreme importance to this purpose and data need to be carefully collected. Globally, updated reports of the largest clinical trials are quite positive, since the persistence of responses observed in B-ALL and B-NHL seems confirmed in the majority of the cases, but follow-up duration remains relatively limited (25, 26, 28).

Importantly, several practical issues need to be considered in order to offer this treatment to all patients who may need it and to integrate CAR T cells in the evolving treatment paradigm of B-cell malignancies.

In the setting of B-ALL, a recent expert opinion from the EBMT and the ASTCT (278) has addressed the possible drawbacks of new treatment options, such as blinatumomab and inotuzumab ozogamicin, and their place in the context of novel cellular therapies. For instance, blinatumomab, which represents an important option for relapsed and MRD positive patients, should be avoided as a bridging therapy before CAR T-cell infusion to minimize the risk of antigen loss (278, 279), although some conflicting data on this issue have been reported (31). The role of allogeneic HSCT after CAR T-cell therapy is being actively debated as well, since some patients (i.e., those with prolonged CAR T-cell persistence and confirmed MRD negative remission) might not need it. The OBERON trial (NCT03628053), a randomized open label multicenter phase III study comparing tisagenlecleucel vs. blinatumomab or inotuzumab in R/R ALL patients, will hopefully better clarify the role of each agent in the treatment paradigm of B-ALL.

In aggressive B-NHL, two CAR T-cell products are now commercially available, and a third one in advanced stage of development (280), thus the question of which one might be preferred will soon be posed. Given the remote possibility of head-to-head randomized clinical trials and the difficulty of driving definitive conclusions from the indirect comparison of the available studies, the issue will likely remain open, with each center experience playing an important role. Furthermore, the role of allo-HSCT in this disease group is probably going to change. Given the relatively limited non-relapse mortality and the fairly good long-term results of CAR T-cell therapy in heavily pretreated R/R B-NHL patients, some authors are now suggesting to employ it earlier in the course of the disease, reserving allo-HSCT for patients who relapse after or who are refractory to CAR T cells (281). Besides, ongoing clinical trials [i.e., BELINDA (NCT03570892), ZUMA-7 (NCT03391466), and TRANSFORM (NCT03575351)] are challenging the place of autologous HSCT, which is being directly tested against CAR T cells, or exploring the use of CAR T cells following autologous HSCT in poor-risk patients (282).

Even in the presence of available commercial products, the enrollment in clinical trials remains essential, both to test newer and hopefully better constructs and to clarify their position in the treatment landscape of each disease. Early referral to centers providing these therapies thus becomes of paramount importance.

Another relevant issue is the affordability of these treatments, especially in the context of public health care systems. As a matter of fact, the total cost for axicabtagene ciloleucel or tisagenlecleucel treatment is close to 1 million dollars per patient, considering the price of the product and all the expenses related to the supportive measures needed (283). Several analyses have been recently presented to address the issue and, albeit quite positive in terms of cost-effectiveness, their conclusions will be confirmed only when mature data on the durability of remission, long-term survival and rate of alloHSCT will be available (283–287). As CAR T-cell therapy becomes available for more indications, the price of the products will hopefully start to be lowered, improving cost-effectiveness and allowing more patients to get access to these treatments. In order to reduce costs, point-of-care production systems, which employ fully automated devices to manufacture CAR T cells, are being tested, possibly reducing the production time as well (288).

After a long road started more than 20 years ago, CAR T-cell therapies have become commercially available. The better understanding of CAR T-cell biology will help to develop strategies to improve their efficacy and safety. New applications in several hematological malignancies and solid tumors are also emerging, but need to be validated by the results of ongoing studies. Nevertheless, translating their application from the small scale of early-phase clinical trials to the large scale of clinical practice will require considerable scientific, logistic, and economic efforts.

## Author Contributions

MCe, MR, M-AP, CV, GP, and BB designed and conceived the review. All the authors collected data. MCe, CV, and DF assembled data. MCe, MR, M-AP, CV, DF, GP, and BB wrote the initial draft of the manuscript. All authors revised and approved the final submitted version of the manuscript.

## Conflict of Interest

MR has received research funding from Novartis; is the inventor in patents involving the use of CART immunotherapy for cancer; and is a consultant/advisor to Nanostring and Abclon. M-AP reports honoraria from Abbvie, Bellicum, Celgene, Bristol-Myers Squibb, Incyte, Merck, Novartis, Nektar Therapeutics, Omeros, and Takeda. He serves on DSMBs for Cidara Therapeutics, Servier and Medigene, and the scientific advisory boards of MolMed and NexImmune. He has received research support for clinical trials from Incyte, Kite/Gilead and Miltenyi Biotec. He serves in a volunteer capacity as a member of the Board of Directors of American Society for Transplantation and Cellular Therapy (ASTCT) and Be The Match (National Marrow Donor Program, NMDP), as well as on the CIBMTR Cellular Immunotherapy Data Resource (CIDR) Committee. GP is employed by Celgene Corporation. The remaining authors declare that the research was conducted in the absence of any commercial or financial relationships that could be construed as a potential conflict of interest.
